# Short-term digital ocular massage may weaken corneal biomechanics

**DOI:** 10.3389/fbioe.2025.1582973

**Published:** 2025-06-18

**Authors:** Andrew K. C. Lam, Sandy N. S. Lee, Mason W. S. Mui, Venus H. Y. Ng

**Affiliations:** ^1^ Centre for Eye and Vision Research, Kowloon, Hong Kong SAR, China; ^2^ School of Optometry, The Hong Kong Polytechnic University, Kowloon, Hong Kong SAR, China; ^3^ Research Centre for SHARP Vision, The Hong Kong Polytechnic University, Kowloon, Hong Kong SAR, China

**Keywords:** Corneal biomechanics, stress strain index, Myopia, massage, intraocular pressure

## Abstract

**Purpose:**

Digital ocular massage has been demonstrated to reduce intraocular pressure (IOP). However, its influence on corneal biomechanics remains unclear. In this study, a device employing Corneal Visualization Scheimpflug Technology (Corvis ST) was used to monitor changes in IOP and corneal biomechanics following short-term digital ocular massage in low and high myopes.

**Methods:**

In total, 29 low myopes and 29 high myopes participated in this study. The right eyes (treatment eyes) underwent digital ocular massage for 5 min, whereas the left eyes (control eyes) remained closed during the procedure. Biomechanically-corrected IOP (bIOP) was measured in both eyes by using Corvis ST at three time points: before the ocular massage, immediately after the ocular massage, and 15 min post-massage. Dynamic corneal response (DCR) parameters were also monitored, namely, peak distance (PeakDist), highest concavity time (HCT), deformation amplitude (DA), deflection amplitude (DefleA), stress-strain index (SSI), time taken to reach the second applanation (A2T), and velocity required to reach the second applanation (A2V).

**Results:**

At baseline, the participants exhibited comparable bIOP in both eyes. A significant reduction in bIOP was observed in the treatment eyes immediately after ocular massage (low myopes: 16.15 ± 2.79 vs. 14.82 ± 3.20 mmHg, p < 0.05; high myopes: 16.81 ± 1.51 vs. 15.39 ± 1.70 mmHg, p < 0.05). Corneal biomechanics at baseline were comparable between the treatment and control eyes. High myopes exhibited more deformable corneas, characterized by a shorter HCT (treatment eyes: 17.30 ± 0.41 vs. 17.72 ± 0.30 msec, p < 0.001; control eyes: 17.33 ± 0.32 vs. 17.55 ± 0.44 msec, p = 0.023), and lower SSI (treatment eyes: 0.739 ± 0.100 vs. 0.848 ± 0.114, p < 0.001; control eyes: 0.741 ± 0.103 vs. 0.858 ± 0.112, p < 0.001) than low myopes at baseline. Immediately after ocular massage, the treatment eyes in both groups exhibited shorter A2T, higher A2V, larger PeakDist, and higher DA and DefleA. Corneal biomechanics in the control eyes remained stable throughout. All DCR parameters returned to baseline levels 15 min after the ocular massage.

**Conclusion:**

Short-term digital ocular massage results in a temporary reduction in bIOP. The observed changes, including shorter A2T, higher A2V, larger PeakDist, and greater DA and DefleA indicated a greater corneal deformability after ocular massage. These findings support the potential association between eye rubbing and the etiology or progression of keratoconus.

## Introduction

Ocular massage is a clinical technique occasionally employed to achieve a temporary reduction in intraocular pressure (IOP). Clinical studies have demonstrated its short-term effectiveness in lowering IOP, making it a valuable intervention in emergency situations or as an adjunct to other treatments. In acute ocular conditions such as angle-closure attack, the gently application of pressure to the closed eyelid with a finger facilitates outflow of aqueous humor, thereby reducing IOP (Razeghinejad et al., 2010). As a non-invasive and immediate intervention, digital ocular massage serves as a practical option when pharmacological treatments are unavailable or while the patient is awaiting a treatment.

Eye rubbing is widely as a critical risk factor in the development and progression of keratoconus (Guo et al., 2023). Numerous studies have reported a higher prevalence of keratoconus among individuals with a history of chronic eye rubbing. The mechanical trauma resulting from frequent and vigorous eye rubbing is believed to damage the corneal epithelium, thereby exacerbating the ectatic process (Jaskiewicz et al., 2023). McMonnies highlighted the correlation between habitual eye rubbing and the onset of keratoconus, suggesting that the mechanical stress disrupts the corneal collagen fibers, ultimately leading to corneal deformation (McMonnies, 2009). Additionally, eye rubbing has been associated with increased levels of matrix metalloproteinases, which are enzymes that degrade collagen, further weakening the corneal structure.

We demonstrated that the reduction in IOP achieved through from digital ocular massage may be partially attributed to the dilatation of Schlemm’s canal (Wu et al., 2024). However, research on the effect of digital ocular massage on corneal biomechanics is limited. Lam and Chen employed an electronic eye massager and reported minimal effects on corneal hysteresis and the corneal resistance factor (Lam and Chen, 2007). By contrast, Liu et al. observed reductions in both corneal hysteresis and corneal resistance factor following eye rubbing (Liu et al., 2011). These parameters measured by the non-contact tonometer, Ocular Response Analyzer (Reichert, Inc., New York, United States) do not represent material properties in conventional physical terms, such as the stress-strain ratio. The Corvis ST (Oculus GmbH, Wetzlar, Germany) is another non-contact tonometer measuring corneal biomechanics by employing an ultrahigh-speed Scheimpflug camera to capture changes in corneal shape during air-puff tonometry. It provides various deformation parameters, including a stress-strain index (SSI), which is a measure of corneal tissue biomechanics. Li et al. employed the Corvis ST and reported a softening effect on the cornea after 1-min of eye rubbing (Li et al., 2023).

This study employed the Corvis ST to assess corneal biomechanics and IOP following short-term digital ocular massage. The measurements were obtained using the Corvis ST before and after the ocular massage intervention.

## Methods

Healthy adult low and high myopes were recruited from a university campus. All the participants underwent a comprehensive eye examination, including slit-lamp biomicroscopy and dilated fundus examination, to rule out ocular diseases. Refractive errors were measured by auto-refraction (Nidek ARK-510A, Nidek Co., Ltd., Japan). Low myopes had spherical equivalent refraction (sphere +½ cylinder power) from −0.50D to −3.00D whereas high myopes had spherical equivalent refraction ≤−6.00D. The inclusion criteria were healthy ocular conditions. Participants with a history of ocular diseases were excluded. This study obtained ethical approval from the university Institutional Review Board (HSEARS20240314003) and the research was conducted in accordance with the tenets of the Declaration of Helsinki. All participants provided informed consent prior to their participation in the study.

Baseline measurements included auto-refraction, keratometry, and axial length (Myopia Master, Oculus Inc., Wetzlar, Germany). The eligible participants were shown an instruction video about digital ocular massage (Hua et al., 2014). The participants performed the ocular massage independently after receiving guidance. They were instructed to close their eyes and use their fingertips to perform the massage (Hafezi et al., 2020). Specifically, they were taught to place their index fingertips on the surface of the upper right eyelid and apply light pressure in a circular motion for 2 s, followed by a 2-s release. This 4-s cycle was repeated continuously for 5 min. The participants were reminded to keep their left eyes closed while massaging the right eye. An examiner supervised the procedure to ensure it was performed correctly throughout the 5 min duration.

### Corvis ST measurement

The Corvis ST is an air puff tonometer equipped with a built-in Scheimpflug ultra-high-speed camera that captures corneal deformation along the horizontal meridian in response to a consistent air pulse. The device records over 4,300 frames per second at high resolution (Supplementary Video S1), enabling the generation of various corneal biomechanics parameters (Tian et al., 2014). Following the air puff, the cornea undergoes inward movement to the first applanation (A1), reaches its highest concavity (HC), and subsequently rebounds to the second applanation (A2) before returning to its original shape. This study focused on selected dynamic corneal responses (DCR) parameters as outlines in Table 1. These parameters include time to reach A1 (A1T), velocity to reach A1 (A1V), time and velocity to reach A2 (A2T and A2V, respectively), deformation amplitude (DA), deflection amplitude (DefleA), peak distance (PeakDist), time to HC (HCT), and stiffness parameter at A1 (SP-A1).

**TABLE 1:** Dynamic corneal response (DCR) parameters.

Parameters	Definition
A1T	Time of the cornea to A1, in millisecond (msec)
A1V	Velocity of the cornea apex reaching A1, in meters/second (m/s)
A2T	Time of the cornea to A2, in millisecond (msec)
A2V	Velocity of the cornea apex reaching A2, in meters/second (m/s)
DA	Sum of pure corneal deflection amplitude and whole eye movement deformation amplitude of the cornea to HC, in mm
HCT	Time of the cornea reaching HC, in millisecond (msec)
PeakDist	Peak distance of the two apices of the cornea at HC, in mm
DefleA	Displacement of the corneal apex in reference to the overlayed cornea from its initial state, in mm
SP-A1	Stiffness parameter at A1, calculated as the adjusted pressure at A1 minus bIOP divided by the deflection amplitude at A1
SSI	Stress-strain index based on numerical modelling input and output parameters CCT, bIOP, and stiffness parameter at HC

A1 = first applanation; A2 = second applanation; HC, highest concavity; bIOP, biomechanically-corrected intraocular pressure.

The stress-strain index (SSI) is an estimate of corneal stiffness that is independent of IOP and central corneal thickness. It is derived from finite element modelling that simulates the corneal response to the air puff generated by the Corvis ST (Eliasy et al., 2019). As a tonometer, the Corvis ST provides both conventional IOP measurements and a biomechanically-corrected IOP (bIOP). The bIOP offers a closer approximation of true IOP, as it is less influenced by corneal biomechanical properties (Eliasy et al., 2018).

Significant reductions in IOP following consecutive Corvis ST measurements have been observed (Borroni et al., 2021). To minimize the potential effects of corneal deformation from the air puff on IOP readings, Corvis ST measurements in this study were taken at three time points: before ocular massage (baseline), immediately after ocular massage, and 15 min post-massage. Only data with an “OK” image quality rating were included in the analysis to ensure repeatability.

### Statistical analysis

All statistical analyses were performed using SPSS (version 26.0, IBM Corporation, Armonk, NY, United States). The Shapiro-Wilk test was applied to test for normality. Continuous variables are presented as mean ± standard deviations. Comparisons between the eyes within each group for age, refractive error, axial length (AL), central corneal thickness (CCT), and bIOP were conducted using either the Student’s t-test or Wilcoxon matched pairs. For comparisons between the right eyes (i.e., treatment eyes) and left eyes (i.e., control eyes) across the two groups at baseline, either the unpaired t-test or the Mann-Whitney test were employed. For gender, a χ^2^ test was used.

Repeated-measures analysis of variance or Friedman tests were used to evaluate changes in CCT and bIOP across the three measurement time points for each eye in each group. Because DCR parameters are influenced by IOP and central corneal thickness, generalized liner models were used to compare corneal biomechanics at baseline and across the three measurement time points for each eye in each group, after adjustment of bIOP and CCT.

The primary outcome measure was DA, which has excellent repeatability and reproducibility (Chen et al., 2014; Bak-Nielsen et al., 2015; Li et al., 2022). Sample size calculations were based on a reported DA of 1.08485 ± 0.102870 mm from over 500 Chinese (Vinciguerra et al., 2022). Assuming a 5% increase in DA following ocular massage, a minimum of 24 subjects was required to achieve 80% power at a significance level of alpha 0.05. Han et al. reported a lower SSI in high myopes (0.813 ± 0.129) compared with low myope (0.920 ± 0.138), with an effect size of 0.801 (Han et al., 2020). To detect a significant difference in SSI between low and high myopes, a minimum of 28 subjects per group was required to achieve 90% power at an alpha 0.05 (G*Power Version 3.1.9.4, Germany).

## Results

A total of 73 participants were initially recruited for the study. Of these, 15 were excluded due to poor image quality obtained from the Corvis ST. Finally, data from 29 low myopes and 29 high myopes were analyzed. Table 2 presents the demographic and ocular characteristics, including age, gender, spherical equivalent refraction (SER), and AL for each group. Within the low myopia group, no significant difference in SER, CCT, and bIOP were observed between the two eyes. However, AL differed significantly between the two eyes (p = 0.025), whereas corneal radii (CRs) was comparable between the two eyes (p = 0.693). By contrast, high myopes exhibited no significant differences in AL or CRs between the two eyes (all p > 0.05). Additionally, both the two myopic groups had similar CRs, CCT, and bIOP in both the treatment eyes (CR: p = 0.870; CCT: p = 0.975; bIOP: p = 0.269) and in the control eyes (CR: p = 0.814; CCT: p = 0.795; bIOP: p = 0.691).

**TABLE 2:** Baseline information of the two myopic groups.

Parameter	Low myopes		High myopes		Significance
Age (years)	21.8 ± 1.2 (19–24)		21.1 ± 2.6 (18–29)		p = 0.008^a^
Gender	13 male vs. 16 female		11 male vs. 18 female		p = 0.594^b^
	Right eye	Left eye	Right eye	Left eye	
SER (D)	−1.71 ± 0.82 (−0.50 to −3.00)	−1.59 ± 0.80 (−0.50 to −3.00)	−8.00 ± 1.31 (−6.25 to −11.25)	−8.00 ± 1.29 (−9.00 to −11.50)	
	p = 0.191^a^		p = 0.968		
AL (mm)	24.28 ± 0.87 (22.30–25.83)	24.19 ± 0.85 (22.21–26.09)	26.76 ± 1.01 (24.78–28.90)	26.69 ± 0.94 (24.80–29.05)	
	p = 0.025		p = 0.371		
CR (mm)	7.80 ± 0.28 (7.28–8.33)	7.80 ± 0.27 (7.33–8.42)	7.79 ± 0.23 (7.38–8.22)	7.79 ± 0.25 (7.39–8.24)	
	p = 0.693		p = 1.000		
CCT (µm)	562.3 ± 31.6 (518.5–631.5)	563.5 ± 29.8 (522.5–640.0)	562.0 ± 26.9 (509.5–610.5)	561.5 ± 29.0 (515.0–626.5)	
	p = 0.206^a^		p = 0.808		
bIOP (mmHg)	16.2 ± 2.8 (11.3–23.3)	16.4 ± 2.7 (12.4–23.7)	16.8 ± 1.5 (14.0–21.0)	16.6 ± 1.5 (13.2–19.3)	
	p = 0.310		p = 0.310		

^a^ non-parametric test.

^b^ Chi square.

SER, spherical equivalent refraction; AL, axial length; CR, corneal radius; CCT, central corneal thickness; bIOP, biomechanically corrected intraocular pressure.

Table 3 presents a comparison of DCR parameters at baseline. Within each myopic group, corneal biomechanics were similar between the treatment and control eyes. However, high myopes had different values compared with the low myopes in some DCR parameters. Specifically, high myopes exhibited faster A2V (treatment eyes: −0.274 ± 0.022 vs. −0.261 ± 0.028 m/s, p = 0.046; control eyes: −0.276 ± 0.023 vs. −0.259 ± 0.029 m/s, p = 0.013) and shorter HCT (treatment eyes: 17.30 ± 0.41 vs. 17.72 ± 0.30 msec, p < 0.001; control eyes: 17.33 ± 0.32 vs. 17.55 ± 0.44 msec, p = 0.023) than those low myopes. Moreover, in both the treatment (0.739 ± 0.100 vs. 0.848 ± 0.114, p < 0.001) and control eyes (0.741 ± 0.103 vs. 0.858 ± 0.112, p < 0.001), high myopes exhibited a lower SSI than those low myopes. Additionally, the treatment eyes of high myopes had a significantly shorter A2T than those low myopes. Conversely, control eyes of high myopes had a significantly longer PeakDist, and greater DefleA than those low myopes.

**TABLE 3:** Generalized liner models were used to compare baseline corneal biomechanics, after adjusting for bIOP and CCT. High myopes demonstrated significantly different corneal biomechanics, in terms of A2Vel, HCT, and SSI, as compared with low myopes. Significant differences were highlighted in red.

Parameter	Low myopes (29)	High myopes (29)	Right eyes (29)	Left eyes (29)
	Right eyes vs. left eyes		Low myopes vs. high myopes	
A1Time *msec*	7.54 ± 0.35	7.61 ± 0.20	7.54 ± 0.35	7.58 ± 0.32
	7.58 ± 0.32 p = 0.696	7.59 ± 0.19 p = 0.680	7.61 ± 0.20 p = 0.384	7.59 ± 0.19 p = 0.896
A1Vel *m/s*	0.139 ± 0.017	0.140 ± 0.014	0.139 ± 0.017	0.140 ± 0.019
	0.140 ± 0.019 p = 0.819	0.142 ± 0.013 p = 0.612	0.140 ± 0.014 p = 0.897	0.142 ± 0.013 p = 0.775
A2Time *msec*	22.28 ± 0.45	22.08 ± 0.26	22.28 ± 0.45	22.26 ± 0.38
	22.26 ± 0.38 p = 0.879	22.13 ± 0.24 p = 0.445	22.08 ± 0.26 p = 0.043	22.13 ± 0.24 p = 0.125
A2Vel *m/s*	−0.261 ± 0.028	−0.274 ± 0.022	−0.261 ± 0.028	−0.259 ± 0.029
	−0.259 ± 0.029 p = 0.773	−0.276 ± 0.023 p = 0.808	−0.274 ± 0.022 p = 0.046	−0.276 ± 0.023 p = 0.013
DA *mm*	1.070 ± 0.103	1.067 ± 0.072	1.070 ± 0.103	1.050 ± 0.104
	1.050 ± 0.104 p = 0.469	1.083 ± 0.077 p = 0.426	1.067 ± 0.072 p = 0.923	1.083 ± 0.077 p = 0.170
HCT *msec*	17.72 ± 0.30	17.30 ± 0.41	17.72 ± 0.30	17.55 ± 0.44
	17.55 ± 0.44 p = 0.081	17.33 ± 0.32 p = 0.755	17.30 ± 0.41 p < 0.001	17.33 ± 0.32 p = 0.023
PeakDist *mm*	4.91 ± 0.25	5.01 ± 0.19	4.91 ± 0.25	4.88 ± 0.25
	4.88 ± 0.25 p = 0.679	5.07 ± 0.21 p = 0.250	5.01 ± 0.19 p = 0.079	5.07 ± 0.21 p = 0.002
*DefleA mm*	0.874 ± 0.089	0.914 ± 0.076	0.874 ± 0.089	0.868 ± 0.090
	0.868 ± 0.090 p = 0.797	0.929 ± 0.076 p = 0.431	0.914 ± 0.076 p = 0.062	0.929 ± 0.076 p = 0.004
SP-A1 *mmHg/mm*	118.03 ± 13.48	123.52 ± 14.75	118.03 ± 13.48	116.76 ± 14.93
	116.76 ± 14.93 p = 0.729	121.23 ± 12.86 p = 0.521	123.52 ± 14.75 p = 0.132	121.23 ± 12.86 p = 0.213
SSI	0.848 ± 0.114	0.739 ± 0.100	0.848 ± 0.114	0.858 ± 0.112
	0.858 ± 0.112 p = 0.730	0.741 ± 0.103 p = 0.950	0.739 ± 0.100 p < 0.001	0.741 ± 0.103 p < 0.001

A1Time, time taken to reach the first applanation; A1Vel, velocity required to reach the first applanation; A2Time, time taken to reach the second applanation; A2Vel, velocity required to reach the second applanation; DA, deformation amplitude of the cornea together with whole eye movement; HCT, time taken to reach the highest concavity; PeakDist, peak distance of the two corneal apices at the highest concavity; DefleA, deflection amplitude of the cornea; SP-A1, stiffness parameter at the first applanation; SSI, stress-strain index.

**FIGURE 1:**
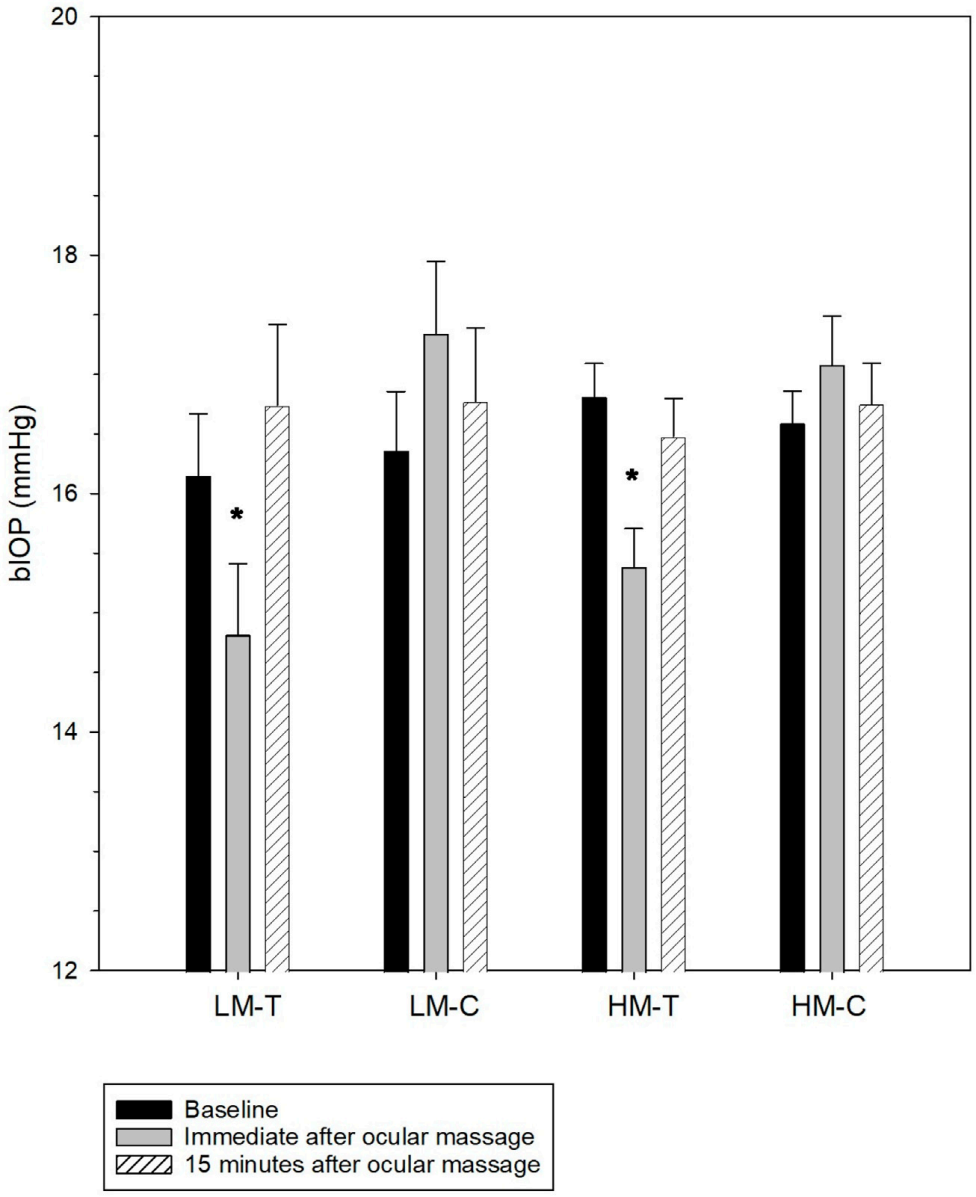
Biomechanically-corrected intraocular pressure (bIOP) at baseline, immediately after, and 15 min after ocular massage. The error bars indicate standard errors. LH-T, treatment eyes of low myopes; LH-C, control eyes of low myopes; HM-T, treatment eyes of high myopes; HM-C, control eyes of high myopes. *significantly different from baseline.

Findings on the effect of ocular massage on corneal biomechanics and IOP are presented in Figure 1 and Table 4. CCT remained stable throughout the experiment in both the treatment and control eyes (all p > 0.05). By contrast, the bIOP of the treatment eyes changed significantly over time (low myopes: Friedman test, p < 0.001; high myopes: repeated measures analysis of variance, p < 0.001), whereas the control eyes maintained stable bIOP levels (all p > 0.05). A *post hoc* analysis revealed significant reduction in bIOP immediately after ocular massage, which returned to baseline levels following a 15-min rest period. After bIOP and CCT were adjusted for, significant changes were observed in some DCR parameters in the treatment eyes of both groups. Conversely, the control eyes exhibited stable corneal biomechanics throughout the experiment. The detailed comparisons of these parameters are provided in Table 4.

**TABLE 4:** Generalized liner models were used to compare corneal biomechanics among baseline, immediately after ocular massage, and 15-min after ocular massage after adjusting for bIOP and CCT. Significant differences were highlighted in red.

Parameter	Low myopes (n = 29)		High myopes (n = 29)	
	Right eye	Left eye	Right eye	Left eye
A1Time *msec*	7.54 ± 0.35	7.58 ± 0.32	7.60 ± 0.20	7.59 ± 0.19
	7.40 ± 0.38 p = 0.090	7.69 ± 0.40 p = 0.490	7.45 ± 0.23^a^ p = 0.013	7.66 ± 0.29 p = 0.509
	7.61 ± 0.45	7.62 ± 0.39	7.56 ± 0.21	7.61 ± 0.25
A1Vel *m/s*	0.139 ± 0.017	0.140 ± 0.019	0.140 ± 0.014	0.142 ± 0.013
	0.147 ± 0.017 p = 0.211	0.135 ± 0.020 p = 0.580	0.148 ± 0.017 p = 0.089	0.139 ± 0.018 p = 0.733
	0.140 ± 0.025	0.138 ± 0.019	0.140 ± 0.015	0.140 ± 0.016
A2Time *msec*	22.276 ± 0.451	22.260 ± 0.381	22.084 ± 0.262	22.134 ± 0.244
	22.508 ± 0.465 p = 0.024	22.085 ± 0.465 p = 0.313	22.299 ± 0.371^a^ p = 0.014	22.028 ± 0.311 p = 0.342
	22.183 ± 0.503	22.194 ± 0.494	22.109 ± 0.292	22.100 ± 0.296
A2Vel *m/s*	−0.261 ± 0.028	−0.259 ± 0.029	−0.274 ± 0.022	−0.276 ± 0.023
	−0.280 ± 0.030 p = 0.039	−0.260 ± 0.033 p = 0.985	−0.290 ± 0.022^a^ p = 0.003	−0.273 ± 0.030 p = 0.913
	−0.265 ± 0.032	−0.259 ± 0.028	−0.270 ± 0.026	−0.276 ± 0.026
DA *mm*	1.070 ± 0.103	1.051 ± 0.104	1.070 ± 0.072	1.085 ± 0.078
	1.113 ± 0.106 p = 0.036	1.025 ± 0.098 p = 0.579	1.136 ± 0.094^a^ p = 0.002	1.078 ± 0.088 p = 0.904
	1.042 ± 0.114	1.045 ± 0.097	1.080 ± 0.070	1.081 ± 0.087
HCT *msec*	17.72 ± 0.30	17.55 ± 0.44	17.30 ± 0.41	17.33 ± 0.32
	17.53 ± 0.44 p = 0.092	17.49 ± 0.42 p = 0.678	17.39 ± 0.59 p = 0.532	17.32 ± 0.37 p = 0.871
	17.54 ± 0.39	17.46 ± 0.43	17.25 ± 0.43	17.37 ± 0.49
PeakDist *mm*	4.91 ± 0.25	4.88 ± 0.25	5.01 ± 0.19	5.07 ± 0.21
	5.06 ± 0.26^a^ p = 0.022	4.82 ± 0.27 p = 0.596	5.15 ± 0.19^a^ p = 0.005	5.02 ± 0.24 p = 0.579
	4.88 ± 0.29	4.87 ± 0.25	5.03 ± 0.18	5.01 ± 0.23
*DefleA mm*	0.874 ± 0.089	0.868 ± 0.090	0.914 ± 0.076	0.929 ± 0.076
	0.939 ± 0.102^a^ p = 0.006	0.844 ± 0.090 p = 0.559	0.975 ± 0.083^a^ p = 0.004	0.909 ± 0.091 p = 0.659
	0.866 ± 0.101	0.861 ± 0.086	0.923 ± 0.069	0.916 ± 0.089
SP-A1 *mmHg/mm*	118.03 ± 13.48	116.76 ± 14.93	123.52 ± 14.75	121.23 ± 12.86
	107.43 ± 15.85^a^ p = 0.002	122.32 ± 12.92 p = 0.276	116.12 ± 15.67 p = 0.155	123.18 ± 15.30 p = 0.743
	119.33 ± 13.92	118.13 ± 14.06	122.32 ± 17.30	120.39 ± 14.96
SSI	0.848 ± 0.114	0.858 ± 0.112	0.739 ± 0.100	0.741 ± 0.103
	0.789 ± 0.131 p = 0.155	0.839 ± 0.115 p = 0.651	0.712 ± 0.104 p = 0.475	0.751 ± 0.110 p = 0.921
	0.821 ± 0.111	0.833 ± 0.099	0.740 ± 0.096	0.751 ± 0.111

^a^ significantly different from baseline.

A1Time, time taken to reach the first applanation; A1Vel, velocity required to reach the first applanation; A2Time, time taken to reach the second applanation; A2Vel, velocity required to reach the second applanation; DA, deformation amplitude of the cornea together with whole eye movement; HCT, time taken to reach the highest concavity; PeakDist, peak distance of the two corneal apices at the highest concavity; DefleA, deflection amplitude of the cornea; SP-A1, stiffness parameter at the first applanation; SSI, stress-strain index.

## Discussion

Our findings revealed that short-term digital ocular massage significantly affected corneal biomechanics, with the effect being transient and observed immediately after ocular massage. After a 15-min rest, the cornea returned to its baseline state. The participants’ left eyes, which served as controls, exhibited stable corneal biomechanics throughout the experiment. At baseline, high myopes exhibited more deformable corneas than low myopes, consistent with previous reports (Han et al., 2020; Liu et al., 2011; Liu et al., 2022; Kenia et al., 2024). For example, the corneas of high myopes had a higher A2V and shorter HCT. Additionally, the corneas of high myopes had a trend of greater PeakDist and larger DefleA than those low myopes (Table 3). These findings may indicate that the corneas of high myopes are more deformable, requiring less time to each the HC and achieving a deeper deformation. Liu et al. suggested that the terms “soft” or “stiff” might not accurately characterize corneal properties (Liu et al., 2024). In this study, we used the term “deformable” (e.g., less deformable or more deformable) to describe corneal biomechanics, and this terminology will be used consistently in the subsequent discussion.

At baseline, the corneas of high myopes exhibited a lower SSI than those low myopes. Kang et al. induced axial myopia in chickens through form-deprivation and observed a reduction in the corneal tangent modulus following axial elongation (Kang et al., 2018). Similarly, Hon et al. employed an indentation method to derive the corneal tangent modulus in humans (Hon et al., 2017) and reported a lower tangent modulus (0.47 MPa) in high myopes than those low myopes (0.57 MPa). Myopia primarily results from axial elongation of the eyeball, as observed in our high myopes (Table 1). Both the cornea and sclera originate from mesoderm, suggesting that measurements of corneal biomechanics could partly reflect scleral biomechanics (Meek and Fullwood, 2001; McBrien and Gentle, 2003). Animal studies have implicated changes in the scleral extracellular matrix components of the sclera in axial elongation (Guggenheim and McBrien, 1996; Harper and Summers, 2015). Alterations in the diameter and density of scleral collagen fibrils are believed to weaken the biomechanical properties of the sclera (McBrien et al., 2001). These biomechanical changes could transmit stretching forces from the sclera to the cornea during axial elongation, thereby influencing corneal biomechanics. A higher A2V (Miki et al., 2017; Yu et al., 2020), and shorter HCT (Yu et al., 2020; Sun et al., 2024) further indicating that the corneas of high myopia are more deformable than the corneas of those low myopia.

DA represents the movement of the cornea along with movement of the whole eyeball when subjected to an air puff. During this process, the eyeball experiences a slightly posterior displacement. By contrast, DefleA isolates corneal deformation by eliminating the influence of the whole eyeball movement, referencing only the corneal profile at its initial stage (Vinciguerra et al., 2016). The treatment and control eyes exhibited similar DA values. However, regarding DefleA, high myopes exhibited higher values in the control eyes (0.929 ± 0.076 mm) compared with low myopes (0.868 ± 0.090 mm, p = 0.004). Similarly, the treatment eyes in high myopes demonstrated higher DefleA (0.914 ± 0.076 mm) than those low myopes (0.874 ± 0.089 mm, p = 0.062), further supporting the notion of a more deformable cornea in high myopia.

Digital ocular massage significantly influenced corneal biomechanics. The treatment eyes in both groups exhibited notable changes in A2T, A2V, PeakDist, DA, and DefleA. Additionally, the treatment eyes in high myopes had a significantly shorter A1T, whereas the treatment eyes in those low myopes had a significantly reduced SP-A1. A shorter A1T, longer A2T, higher A2V, larger PeakDist, greater DA and DefleA, and lower SP-A1 following ocular massage collectively indicate increased corneal deformability (Table 3). The cornea is not a purely elastic material but exhibit viscoelastic properties. Parameters such as A1T, A2T, and A2V may reflect the corneal viscoelasticity, whereas PeakDist and DefleA represent its elastic response under air pressure (Ali et al., 2014; Liu et al., 2024). We found that short-term ocular massage resulted in a weaker, more deformable cornea.

Lam and Chen, who employed an electronic eye massager, did not report significant changes in corneal hysteresis (Lam and Chen, 2007). Liu et al. reported a decrease in corneal hysteresis following eye rubbing, which was performed by the participants themselves (Liu et al., 2011). Corneal hysteresis reflects the viscoelastic properties of the cornea, and the observed decrease was attributed to a reduction in the viscosity of proteoglycans, which bind collagen fibrils within the cornea. McMonnies et al. employed an optical pachometer and reported a considerable thinning of the corneal epithelium resulting from eye rubbing (McMonnies et al., 2010). However, this finding was not corroborated by Kalogeropoulos et al. (Kalogeropoulos et al., 2009). Prakasam et al., who employed spectral-domain optical coherence tomography, also failed to identify significant changes in the corneal epithelium and Bowman’s layer (Prakasam et al., 2012). These discrepancies may be attributed to variations in the rubbing forces applied across studies. Notably, Pollmann et al. reported a case of corneal epithelial thickening and hypertrophy due to chronic eye rubbing, which was reversible upon cessation of the rubbing behavior (Pollmann et al., 2024).

Corneal stiffness, as measured using SP-A1, was reduced after eye rubbing in myopes but not in emmetropes (Li et al., 2023). It potentially reflected baseline differences in corneal biomechanics between myopes and emmetropes (Lee et al., 2016; He et al., 2017). Although Li et al. used a similar circular motion as in the current study, Their protocol involved a single examiner applying pressure with the knuckle of their index finger for 1 min (Li et al., 2023). They reported considerable reduction in CCT, whereas no significant change in CCT was observed in either the treatment and control eyes in our study. Notably, our participants exhibited changes in corneal biomechanics only in the treatment eyes, but not in the control eyes, confirming that short-term ocular massage can transiently modify the viscoelastic and elastic properties of the cornea. The observed changes in corneal biomechanics were temporary and returned to baseline levels shortly after the massage. This finding is consistent with that reported by Liu et al. (2011) which suggested that corneal hysteresis required over 2 min to recover to baseline levels following an eye-rubbing episode. However, unlike those of previous studies, our findings did not indicate changes in SP-A1 following ocular massage (Li et al., 2023). They noted that younger patients were more susceptible to changes induced by eye rubbing. Both SP-A1 and SSI represent material properties of the cornea, and appeared stable and unaffected by short-term ocular massage in our cohort. The long-term effects of prolonged or repetitive ocular massage remain unknown. Persistent or vigorous ocular massage or rubbing could lead to adverse biomechanical changes over time. Thus, patients should be advised to avoid such behaviors to prevent potential harm to the cornea.

Eye rubbing has been strongly associated with keratoconus (Najmi et al., 2019; Sahebjada et al., 2021), particularly in severe cases (Guo et al., 2023). Guo et al. identified eye rubbing as a potentially overlooked risk factor for keratoconus among Chinese (Guo et al., 2023). Notably, the patients in Guo et al. frequently rubbed their eyes using their knuckles (Guo et al., 2023), which can exert significantly greater force compared with using fingertips (Hafezi et al., 2020). Eye rubbing should be discouraged due to the potential damage caused by this behavior to the corneal epithelium, where it can induce cell apoptosis, disrupt cell adhesion, and trigger cellular stress (Jaskiewicz et al., 2023). Furthermore, an increased in tear matrix metalloproteinases (e.g., MMP-1 and MMP-13) and inflammatory mediators following eye rubbing has been observed in patients with keratoconus, which may be associated with the progression of keratoconus (Balasubramanian et al., 2013).

We utilized 15 min of digital ocular massage and the effect was transient. Eye care practitioners should enquire about eye rubbing habits, particularly in patients with ocular allergies (Dimacali et al., 2020) or keratoconus (Guo et al., 2023). Liu et al. conducted an *in vitro* study utilizing rabbit corneal fibroblasts (Liu et al., 2014). Mechanical stress stimulated the synthesis of corneal matrix metalloproteinases and the activation of extracellular controlled protein kinases. Comparable results were observed in human corneal fibroblasts, attributed to changes in cell shape and function from mechanical stress (Zhang et al., 2021).

Given the impact of mechanical stress on corneal cells and the corneal cellular matrix, it is essential to assess for eye rubbing especially relevant for subclinical keratoconus patients or individuals undergoing evaluation for refractive surgeries. As D'Oria et al. emphasized, early detection and biomechanical assessment are critical in refractive surgery planning (D'Oria et al., 2024). Even transient biomechanical weakening—such as that observed after short-term digital ocular massage—could have compounded effects in patients with borderline corneal stability. Thus, patient education regarding the potential risks of habitual eye rubbing is vital. Preventive strategies, including the discouragement of frequent eye rubbing and comprehensive biomechanical screening, should be incorporated into standard pre-operative protocols.

A significant IOP reduction following ocular massage was observed, consistent with findings from our previous study. The regulation of IOP is primarily determined by aqueous humor production and its outflow. The majority of the aqueous humor drains through the trabecular meshwork into the lumen of the Schlemm’s canal. It drains into aqueous veins and episcleral veins via collector channels eventually, which constitutes the conventional pathway (Carreon et al., 2017). A small fraction of aqueous humor drains via uveoscleral outflow, known as the unconventional pathway (Schmidl et al., 2015). Wu et al. reported an IOP reduction from 15.7 ± 2.5 mmHg at baseline to 9.6 ± 2.2 mmHg immediately after 10 min of ocular massage (Wu et al., 2024). Even at five minutes after ocular massage, the IOP remained significantly below the baseline level. Through anterior segment optical coherence tomography, they demonstrated dilatation of Schlemm’s canal following ocular massage, suggesting that the observed IOP reduction could be partially attributed to enhanced aqueous outflow. However, their study did not monitor IOP until it returned to baseline levels. In the present study, bIOP returned to baseline levels after a 15-min rest period. Although Wu et al. applied ocular massage for 10 min, which may have resulted in a more substantial IOP reduction (Wu et al., 2024), their study relied on rebound tonometry, a method that can be influenced by corneal biomechanics (Chui et al., 2008). If the cornea is weakened following ocular massage, lower applanation force is required to achieve the tonometry end point, potentially leading to an underestimated IOP measurement. In this study, we applied bIOP, a measure that accounts for both corneal dimensions and biomechanical properties. Our findings confirm that ocular massage has a measurable effect on IOP reduction.

The current study has some limitations. A major challenge was the variability in the intensity of ocular massage, which was difficult to standardize. The participants were instructed to massage their eyes gently to avoid discomfort. The use of electronic ocular massager could provide more consistent force across participants and reduce this variability (Lam and Chen, 2007). Although bIOP offers an IOP independent of corneal properties, including corneal thickness, our frequency of IOP measurements was insufficient to monitor changes in IOP during the 15-min rest period. The final measurements were conducted 15 min after digital ocular massage. In our previous study, we found that rebound tonometry was still significantly lower than the baseline level at 5 min after digital ocular massage. Thus, we assumed that 15 min might be enough for the bIOP to return to the baseline level. We did not measure IOP frequently due to the notion that frequent tonometry from the Corvis ST may overly influence the IOP readings (Borroni et al., 2021). To maintain consistency, the fellow eye was kept closed, ensuring that only the treatment eye was subject to ocular massage. We intended to massage the central cornea as much as possible. Participants were reminded to maintain a steady central fixation even with their eyes closed. Al alternative approach could have been to keep the fellow eye open, which may have better controlled for steady central fixation (Prakasam et al., 2012; Balasubramanian et al., 2013). Wu et al. reported an association between IOP reduction and dilatation of Schlemm’s canal following ocular massage (Wu et al., 2024). Anterior segment optical coherence tomography could be employed in future studies to monitor changes in anterior ocular structures, providing further insights into aqueous humor dynamics. Only young Chinese adults were included in the current study. Our results may not be generalized to other ethnic and age groups.

In conclusion, corneas of high myopes exhibit greater deformability than those low myopes. Short-term ocular massage leads to a reduction in IOP, and induces certain changes in corneal biomechanics, affecting both low and high myopes. However, 15 min of digital ocular massage is different from chronic eye rubbing. Further research is required to investigate the long-term effects of ocular massage or rubbing on corneal biomechanics and its potential association with keratoconus development.

## Data Availability

The raw data supporting the conclusions of this article will be made available by the authors, without undue reservation.
